# Leveraging Single-Case Experimental Designs to Promote Personalized Psychological Treatment: Step-by-Step Implementation Protocol with Stakeholder Involvement of an Outpatient Clinic for Personalized Psychotherapy

**DOI:** 10.1007/s10488-024-01363-5

**Published:** 2024-03-11

**Authors:** Saskia Scholten, Lea Schemer, Philipp Herzog, Julia W. Haas, Jens Heider, Dorina Winter, Dorota Reis, Julia Anna Glombiewski

**Affiliations:** 1 Department of Psychology, Pain and Psychotherapy Research Lab, RPTU Kaiserslautern-Landau, Ostbahnstr. 10, 76829 Landau, Germany; 2 Department of Psychology, Harvard University, 33 Kirkland Street, Cambridge, MA 02138 USA; 3 Applied Statistical Modeling, Universität des Saarlandes, Campus, 66123 Saarbrücken, Germany

**Keywords:** Single-case experimental design, Experience sampling method, Ecological momentary assessment, Personalized psychotherapy, Stakeholder involvement, Implementation research

## Abstract

Our objective is to implement a single-case experimental design (SCED) infrastructure in combination with experience-sampling methods (ESM) into the standard diagnostic procedure of a German outpatient research and training clinic. Building on the idea of routine outcome monitoring, the SCED infrastructure introduces intensive longitudinal data collection, individual effectiveness measures, and the opportunity for systematic manipulation to push personalization efforts further. It aims to empower psychotherapists and patients to evaluate their own treatment (*idiographic perspective*) and to enable researchers to analyze open questions of personalized psychotherapy (*nomothetic perspective)*. Organized around the principles of agile research, we plan to develop, implement, and evaluate the SCED infrastructure in six successive studies with continuous stakeholder involvement: In the project development phase, the business model for the SCED infrastructure is developed that describes its vision in consideration of the context (Study 1). Also, the infrastructure's prototype is specified, encompassing the SCED procedure, ESM protocol, and ESM survey (Study 2 and 3). During the optimization phase, feasibility and acceptability are tested and the infrastructure is adapted accordingly (Study 4). The evaluation phase includes a pilot implementation study to assess implementation outcomes (Study 5), followed by actual implementation using a within-institution A-B design (Study 6). The sustainability phase involves continuous monitoring and improvement. We discuss to what extent the generated data could be used to address current questions of personalized psychotherapy research. Anticipated barriers and limitations during the implementation processes are outlined.

**Supplementary Information:**

The online version contains supplementary material available at 10.1007/s10488-024-01363-5.

## Introduction

Clinicians face the challenge that they work with individuals, while most research is still conducted on the group level. Nomothetic research was intended to identify general psychological processes (Allport, 1937). However, even relatively homogenous groups are composed of heterogeneous individuals. Trying to understand intraindividual variation inferring from results based on interindividual differences may lead to invalid conclusions (Molenaar, 2004). In psychotherapy research, heterogeneity of treatment effects (Varadhan et al., 2013) indicates that patients respond differently to psychological treatments even if they show similar symptoms and are treated by the same therapist with similar methods (Kiesler, 1966). Recently, meta-analyses empirically demonstrated the considerable heterogeneity in treatment effects for several mental disorders such as depression (Kaiser et al., 2022a, 2022b), posttraumatic stress disorder (Herzog & Kaiser, 2022) and borderline personality disorder (Kaiser & Herzog, 2023) with small to medium effects on average depending on the mental disorder studied. These findings underly the need for optimization efforts to maximize treatment outcomes of the individual patient. In this regard and analogous to personalized medicine (Simon & Perlis, 2010), personalizing psychological treatments to the individual patient might be a promising approach to achieve this goal (Chekroud et al., 2021).

### Personalized Treatment Approaches

Personalized psychological treatments aim at investigating the dynamics in psychopathology (Fisher, 2015) and mechanisms of change within an individual patient over the course of treatment (Altman et al., 2020). Generally speaking, mental disorders are considered complex systems of contextualized dynamic processes that are specific to the individual and need to be considered in deriving personalized treatment decisions and recommendations (Wright & Woods, 2020).

Personalized treatment approaches can be based on intuitive, theoretical, data-informed, or data-driven models (Cohen et al., 2021). Sources of information for clinical decision-making are intuition and anecdotes for intuitive models, and theory and concepts for theoretical models, respectively (Cohen et al., 2021). Yet, the resulting clinical judgements may be error-prone due to several clinician biases such as representativeness heuristic, selection, and confirmation biases, as well as overoptimism (Lutz et al., 2022). In contrast, data-informed and data-driven models are based on evidence or statistical algorithms (Cohen et al., 2021). One data-based line of research led to the development of empirically derived decision-support tools such as systematic routine outcome monitoring (Lutz et al., 2022). Objective feedback allows individuals to form accurate pattern-recognition abilities (Kahneman & Klein, 2009). Another line of research comprises treatment selection procedures based on clinical prediction models. They aim to achieve a better match between the individual and the treatment received (Cohen & DeRubeis, 2018). Both approaches are highly valuable to address clinician biases, as a complementary tool, and could yield a potential to increase treatment outcomes. Limitations to these data-informed approaches include that many studies are underpowered or lack hold-out samples and are therefore likely to overestimate effects of personalized treatment (Lorenzo-Luaces et al., 2021). In addition, routine outcome monitoring is currently observational, raising questions about the internal validity, and thus limiting the causal inferences (Kaiser et al., 2023). Prediction models are also limited due to their reliance on group level information and large datasets which are not feasible to collect in routine clinical care and private practices.

To push personalization efforts even further, clinician scientists or scientific practitioners need to be empowered to implement successful personalized psychological treatment (Piccirillo & Rodebaugh, 2019). An experimental setting within general practice that focuses on the individual patient is needed (Howard et al., 1996). New technologies, such as experience sampling methods, wearables, open-source software for analyzing time-series data, and more advanced statistical models could assist the integration of individual level designs into clinical practice (Piccirillo & Rodebaugh, 2019).

### Promoting Idiographic Research with Single-Case Designs

Single-case experimental designs (SCEDs^1^) are time- and cost-efficient, yet methodologically sound, prospective idiographic research designs (Nikles et al., 2021) that could be particularly promising to be implemented in routine clinical care (Schemer et al., 2022). Per definition, SCEDs have the following key features: (1) One entity (e.g., a person) that serves as its own control, (2) is observed repeatedly during a certain period of time (for example via experience sampling methodology, ESM^2^), and (3) under systematic manipulation (e.g., introduction of a psychological treatment) (Vlaeyen et al., 2022). SCEDs seek to estimate meaningful treatment effects for individual patients while safeguarding internal validity and causal inference (Kazdin, 2019; Tanious & Onghena, 2019; Vlaeyen et al., 2020). In combination with ESM, the methodological rigor of SCEDs can be further increased (Schemer et al., 2022). Graphical examples of SCEDs are presented as Supplemental Material (S1 Design illustrations, https://osf.io/dkytg).

SCEDs, especially in combination with ESM, are considered powerful and adaptive research designs for routine clinical care that could reduce the research-practice gap (Bentley et al., 2019; Berg et al., 2023; Kravitz et al., 2014). Both methodological approaches bring different advantages. ESM has shown to support clinical care usefully (e.g., problematic cannabis use: Piccirillo et al., 2023)**.** In particular, both researchers and practitioners highlight that ESM has an incremental value for mental health care, with the assessment of context specificity of symptoms as the most useful part (Piot et al., 2022). SCEDs on the other hand allow to evaluate the efficacy of existing interventions or intervention packages for a particular patient in clinical practice and to easily pilot novel interventions or modifications of known interventions (Krasny-Pacini & Evans, 2018; Selker et al., 2022).

Existing evidence indicates that 79% of participating patients found n-of-1 trials useful (Kaplan & Gabler, 2014) with high perceived system usability (Kravitz et al., 2020). In a medical context, SCEDs are used for example to study the ideal dose of melatonin for sleep disturbance in Parkinson's disease in a way that individual study participants also gained valuable insights (Nikles et al., 2019). Likewise, SCEDs are also particularly suitable for treating mental health problems lacking (adequate) evidence for the efficacy of specific psychological treatments (Duan et al., 2013). Due to high comorbidities and the fact that individuals are not alike, it is often the case that existing protocols are not suitable for patients in routine clinical practice (Berg et al., 2023). By quickly identifying ineffective psychological treatments, SCEDs can help to reduce mental health care costs (Kravitz et al., 2014). In addition, patients may be involved more closely in treatment planning through self-monitoring (Kravitz et al., 2014) and shared decision-making is encouraged through continuous discussion (Riese et al., 2021). The result is a patient-centered, genuine learning system in mental health care (Selker et al., 2022).

In practice, this means that the effects of specific interventions carried out by a practitioner on a particular patient could be evaluated throughout the ongoing treatment (mind: Paul, 1967). A SCED infrastructure allows to set up research studies more easily. For example, a multiple baseline design could be implemented to evaluate isolated intervention strategies (Schemer et al., 2018). Collected data could then be analyzed on an individual level (e.g., single-case randomization tests) and on a group level (e.g., single-case meta-analysis) (Heyvaert & Onghena, 2014).

### Objective

Our ultimate objective is to establish an Outpatient Clinic for Personalized Psychotherapy within a German outpatient research and training center. Its key component is a SCED infrastructure creating an experimental setting centered on individual patients within routine clinical care serving the following aims:

1. *Idiographic perspective:* Enriching current diagnostic procedures mainly comprising clinical interviews and questionnaires by ESM methods that allow continuous monitoring of relevant psychological processes.
2. *Causal inference:* Facilitating systematic manipulation going beyond current standards of routine outcome monitoring systems to find effective treatments for challenging or non-responsive patients and to optimize new psychological treatments for future studies.
3. *Nomothetic perspective:* Over time, as data accumulates, large numbers of individual intensive-longitudinal datasets could be used for data-driven personalization efforts.

To achieve these long-term goals, we plan to develop, implement, and evaluate an SCED infrastructure following agile research principles (Wilson et al., 2018a). The current paper serves as a study protocol for this step-by-step implementation process. The infrastructure serves two areas of application: routine clinical care and future research projects. While this study protocol outlines the planned procedure in routine clinical care, future research projects may adapt their methodological approaches. Unlike typical implementation research focused on effective interventions (Pinnock et al., 2017), we plan to implement a methodological infrastructure. Reporting standards for implementation studies recommend a twofold reporting strategy always reporting on the implementation strategy (e.g., training of therapists) and the intervention (e.g., exposure therapy). Yet, the SCED infrastructure is not seen as an intervention. Therefore, we focus on typical implementation outcomes such as feasibility, acceptability, and sustainability of the infrastructure (Proctor et al., 2011) as oppose to its effect on clinical outcomes.

## Methods and Analysis

To promote an open research culture (Nosek et al., 2015), an Open Science Framework (OSF) project is created to bundle the projects that will be realized using the SCED infrastructure, including respective pre-registrations using the SCED infrastructure, research materials, and preprints (https://osf.io/yex48/).

### Setting

The SCED infrastructure will be implemented in the outpatient clinic of the RPTU Kaiserslautern-Landau in Germany. The outpatient clinic is part of a project for coordinating research efforts in German university outpatient clinics for psychotherapy that guides current standard diagnostic procedures (Velten et al., 2017). Standard diagnostic procedures comprise up to four diagnostic sessions and include an intake interview by a licensed psychotherapist, further clinical interviews and questionnaires. The Symptom Checklist 90 (SCL-90, Derogatis & Unger, 2010; Franke, 2014) together with symptom-specific questionnaires is administered at intake, during the diagnostic sessions, during treatment (every 10th session), at posttreatment and at 6-month follow-up.

Around 800 adults with mental disorders are treated per year. Eighty therapists in training and 18 licensed psychotherapists deliver about 45 treatments per day. In Germany, public health insurance usually covers short-term treatment of 24 sessions, which can be extended to up to 80 sessions in total, depending on the mental state of patients. In addition, therapies are implemented within the framework of third-party funded and self-funded psychotherapy studies. The treatment delivered is Cognitive Behavioral Therapy (CBT). The outpatient clinic is a facility that teaches bachelor's graduates who are pursuing their master's degree in psychotherapy and master's graduates who are training to become licensed psychotherapists with a special emphasis on CBT. Adherence to standard CBT methods is safeguarded by regular supervision. Bachelor graduates receive intensive one-on-one supervision by a licensed psychotherapist, who could directly intervene in the case of an emergency, while working with patients. Master graduates receive regular group and individual supervision by a licensed psychotherapist with more than five years of work experience.

### Identification of Stakeholders

The following stakeholders have to be considered during the implementation process (Eslick & Sim, 2014; Krasny-Pacini & Evans, 2018; Selker et al., 2022): *Patients (and their family members, caregivers…)* have to be able to easily enter and access their own data and to view and interpret their results. *Therapists (and their supervisors)* should be able to recruit and manage a sample of patients, to set up their own SCDs, monitor the data collection progress, intervene if needed, and to view and interpret results. *Clinical researchers* should be able to create and implement SCEDs, intervene if needed, capture deviations and adjust the protocol, and assess fidelity measures and interrater-reliability. The *administrative team* provides institutional oversight and management (e.g., creating user accounts). *System administrators and developers* support the operation of the IT system, provide user tech support, and maintain and problem-solve the operational code. The *statistician* reviews the trial design and collected data for validity and/or aggregate analysis, download identified or de-identified data for offline analysis, and runs the analysis. Other potentially relevant stakeholders are *healthcare payers, healthcare delivery systems, and regulatory agencies.* In addition, experts in the field form the *scientific advisory board* that supervises the implementation of the Outpatient Clinic for Personalized Psychotherapy and *training staff* of the psychotherapy training consider SCD and m-Path in the curriculum to enable future therapists to easily use the methodology. Stakeholders and their key functions for the SCED infrastructure are summarized in the Supplemental Material (S2 Stakeholder identification, https://osf.io/z4uf5).

### Implementation Strategy: Step-by-Step Implementation with Stakeholder Involvement

A successful implementation of the SCED infrastructure needs to emphasize the context where the SCED infrastructure is introduced (Bauer & Kirchner, 2020). Implementation strategies are methods or techniques used to enhance the adoption, sustainability, and scaling up (Powell et al., 2015; Proctor et al., 2013). Discrete implementation strategies involve single approaches or techniques, while the complexity of implementing clinical innovations often necessitates multifaceted strategies that combine two or more of these discrete strategies (Kirchner et al., 2020a). Implementation strategies should be considered from the outset of the planned research (Pinnock et al., 2017), even though they are iterative by nature and need to be adapted when facing unexpected challenges (Kirchner et al., 2020a). We plan to use a step-by-step implementation with stakeholder involvement as multifaceted implementation strategy (compare “stage implementation scale up” and “using advisory boards and workgroups” as recommended implementation strategies by the Expert Recommendations for Implementing Change (ERIC) project; Powell et al., 2015).

**Fig. 1:**
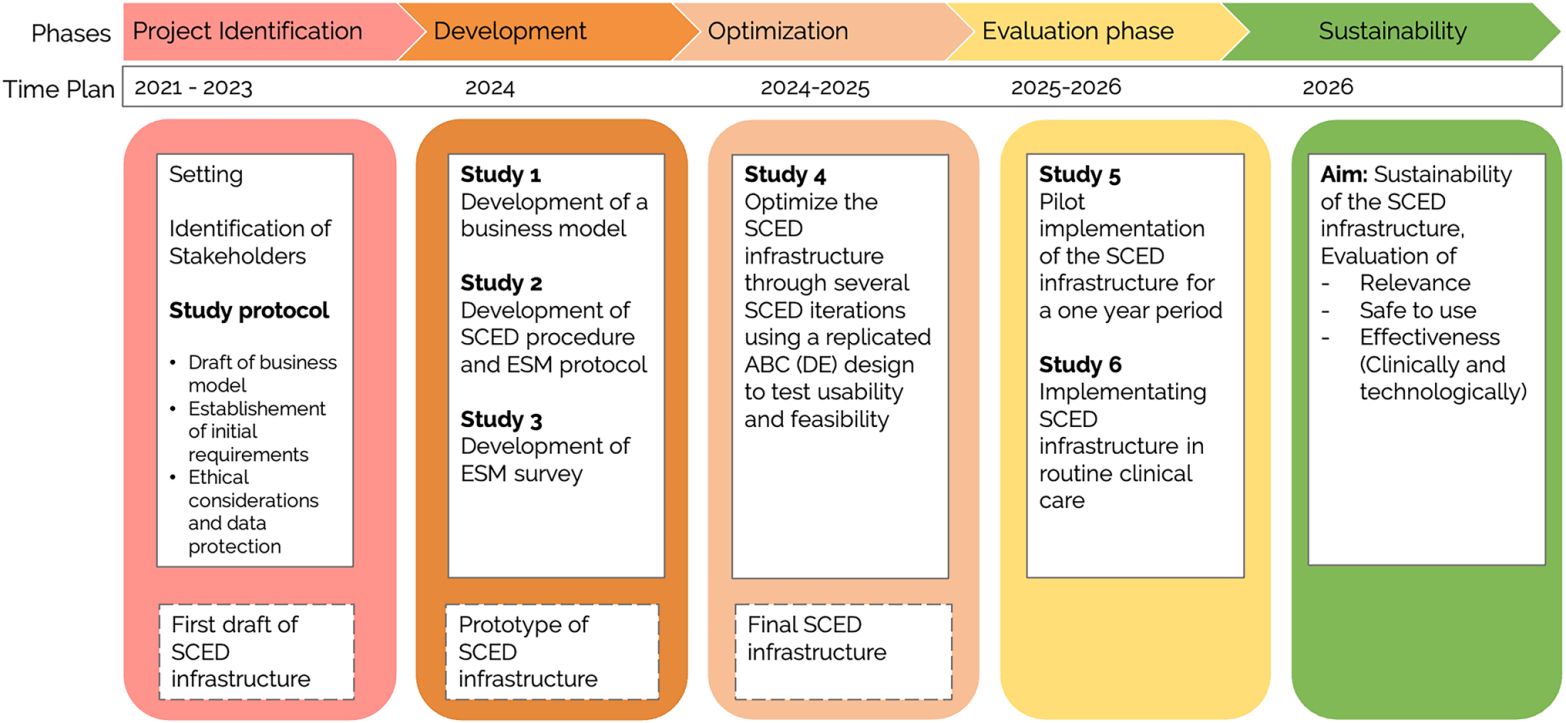
Step-by-step implementation process

Following the recommendations of agile research (Bartels et al., 2022; Wilson et al., 2018), different steps in the implementation process can inform and potentially modify subsequent steps (Fig. 1): In *the project identification phase*, the SCED infrastructure is envisioned (see Objectives) while acknowledging the context of the SCED infrastructure (see Setting and Stakeholder Identification). It is dedicated to plan the step-by-step implementation and to describe it in this study protocol. In *the project development phase,* a prototype of the infrastructure is specified. This includes finalizing a business model in consideration of the context (Study 1), the SCED procedure, ESM protocol and ESM survey (Study 2 and 3). In *the optimization phase*, feasibility and acceptability are tested and the infrastructure is customized accordingly (Study 4). The *evaluation phase* includes a pilot implementation study to assess implementation outcomes (Study 5) and the actual implementation using a within-institutional A-B design to evaluate the implementation (Study 6). In *the sustainability phase,* the application is continuously monitored and improved regarding relevance, safety, and effectiveness. Throughout the entire process, we will establish and consult an advisory board consisting of international experts in SCEDs that meets twice a year to guide project decision making.

Stakeholders will serve as research partners (Anampa-Guzmán et al., 2022) who will be consulted at several stages through various methods (e.g., via stakeholder meetings and interviews) to develop, optimize, and implement the SCED infrastructure. This involvement provides stakeholders with the opportunity to express what their group needs during the implementation process (such as special trainings or workshops). As outlined above, we consider different types of stakeholders: patients, therapists, supervisors, and clinical researchers as *users* of the SCED infrastructure; statisticians, administrative staff, and developers as *user support* (e.g., administrators, developers), and the scientific advisory board and our training staff as *collaborators*. Stakeholders are engaged using various methods (see separate pre-registrations for detailed descriptions of the respective methods) such as the World Café Method (Brown & Isaacs, 2005; Schiele et al., 2022), stakeholder meetings (Doria et al., 2018), cognitive interviews (Beatty & Willis, 2007), and semi-structural interviews (Domecq et al., 2014). Involvement will be evaluated using the Public and Patient Engagement Evaluation Tool (PPEET) (Abelson et al., 2016) and reported according to the GRIPP2 checklist (Staniszewska et al., 2017).

### Study 1: Exploring Barriers and Facilitators to Develop a Business Model

The aim of this study is (1) to explore barriers and facilitators regarding the prospective implementation process and (2) to develop a business model that enables sustainable implementation. For this purpose, a preliminary business model is being formulated, initial technical requirements are being considered, and ethical and privacy issues are being assessed to identify potential questions and challenges. We will conduct a World Café (Brown & Isaacs, 2005; Schiele et al., 2022) at least three persons of all relevant stakeholder groups (patients, therapists, supervisors, researchers) to identify further questions and challenges. In qualitative research, information saturation in interview studies is reached after 6–15 interviews (Guest et al., 2006; Turner-Bowker et al., 2018). Because recommendations for group sizes in World Cafés are missing, we aimed at a group size of 12 persons based on this information. The World Café is followed by in-depth stakeholder interviews with key experts for specific questions (e.g., regarding data protection, psychotherapy training) to develop a business model that addresses these issues.

#### Draft of the Business Model

**Fig. 2:**
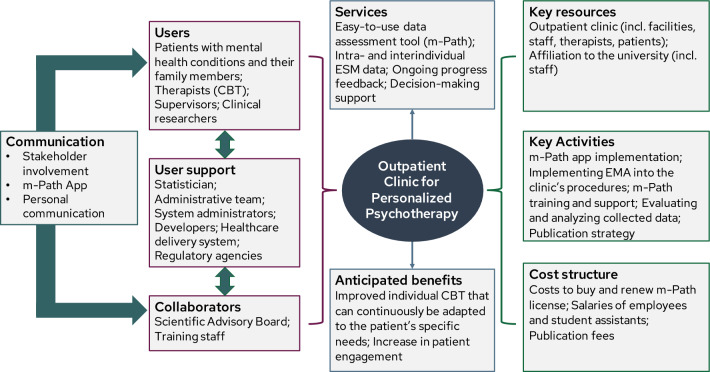
Draft of the Business Model of the Outpatient Clinic for Personalized Psychotherapy

The business model is intended to describe how the Outpatient Clinic for Personalized Psychotherapy will create, deliver, and capture value (Osterwalder et al., 2010). Business models have a long tradition in entrepreneurial practice and have been recommended for clinical trials (McDonald et al., 2011) and health care service planning (Sibalija et al., 2021). Developing a business model obliges to think through economic, operational, and strategic aspects that will lead to sustainable benefits in defined markets such as health care services (Morris et al., 2005). Figure 2 shows the first draft of the business model we developed for the Outpatient Clinic for Personalized Psychotherapy following Osterwalder and colleagues' nine building blocks (2010). Subsequently, we will outline each block and highlight questions and challenges (Q&C) that need to be clarified in the development phase:

1. The Outpatient Clinic for Personalized Psychotherapy aims to reach and serve patients with mental health conditions and their family members, therapists in training and licensed therapists, their supervisors, and clinical researchers (called “*users”,* see “stakeholders” for more information). Q&C: *How interested are these users in the Outpatient Clinic for Personalized Psychotherapy? What are the incentives and the support needed to use the Outpatient Clinic for Personalized Psychotherapy?*
2. The Outpatient Clinic for Personalized Psychotherapy provides the following *services* that create value for the users: an easy-to-use data assessment tool, intra- and interindividual ESM data, ongoing progress feedback, and data-based clinical decision-making support. Q&C:* What is needed to make the service (organizationally and technically) easily accessible? How should it be set up that it is accepted and feasible?*
3. *Communication or channels* connect the users with the services within the Outpatient Clinic for Personalized Psychotherapy. They include the research co-design process to raise awareness and evaluate the concept of the Outpatient Clinic for Personalized Psychotherapy, the ESM app m-Path to use and deliver the services, and personal communication to provide support and to stay in touch (Mestdagh et al., 2022). Q&C:* How are different channels connected to each other, e.g., how is feedback provided within m-Path or to a particular therapist channeled to the administrative staff or the research team?*
4. Types of relationships between the Outpatient Clinic for Personalized Psychotherapy and its users (formerly: customer relationships) range from personal assistance to automated services. For a successful implementation, the Outpatient Clinic for Personalized Psychotherapy needs a *user support* system that includes a statistician, an administrative team, system administrators, developers, the healthcare delivery system, and regulatory agencies (see “stakeholders” for more information). Q&C: *How can the Outpatient Clinic for Personalized Psychotherapy be embedded into the existing routine clinical care structure and procedures?*
5. *Anticipated benefits* are improvements of personalized psychological treatments that can continuously be adapted to the patient’s specific needs and an increase in patient engagement. These benefits are intended to produce reduced mental health care costs in the long run allowing to negotiate with healthcare insurances to pay for the service. Q&C:* How can empirical evidence be provided that examine these anticipated benefits? What are the relevant outcomes?*
6. *Key resources* describe the most important assets (physical, human, financial, and intellectual) that are required to make the business model work. The outpatient clinic is a major asset because it provides the facilities, but also the infrastructure of the healthcare provision including patients, therapists, supervisors, and staff members, likewise generating financial resources. The affiliation to the university is also a key resource because it includes clinical researchers, student assistants, and an intellectual network, e.g., to the scientific advisory board. Q&C: *How the Outpatient Clinic for Personalized Psychotherapy integrated into the existing workflow and -packages to ensure long-term implementation?*
7. *Key activities* that are needed to make the business model work include the implementation of a platform that allows to collect and visualize ESM data, the evaluation and analysis of the collected data, and the publication strategy. Q&C: *Which platform should be selected? How are workflows covered that are not implemented in the platform yet?*
8. *K*ey partnerships or *collaborators* (see “stakeholders” for more information) make the business model work. Q&C: *How are stakeholders selected, engaged, and continuously involved into the process?*
9. The *cost structure* describes all the costs incurred to operate the business model. In our case, the costs to buy and maintain the license for the platform, the salaries of the employees and student assistants, and, if needed, publication fees. Currently, all costs are covered by the university and the outpatient clinic. Q&C: *How can costs be covered in the long run to guarantee sustainable funding for the Outpatient Clinic for Personalized Psychotherapy?*

### Establishment of Technical Requirements

The Outpatient Clinic for Personalized Psychotherapy's key service is a platform facilitating all SCED phases: goal selection, treatment, measures, trial design (analysis, sampling, randomization, blinding, scheduling, and reviewing), data collection, and analysis (Eslick & Sim, 2014). Frequent graphing of data and adaptability to new information are crucial (Hayes, 1981). To facilitate shared decision-making, data presentation (e.g., treatment effect size, confidence intervals, graphical trajectories, means) must avoid overinterpretation, incorporate user preferences, and maintain scientific rigor (Duan et al., 2013). Using Eslick and Sim's (2014) checklist for platform selection, we decided to use m-Path (Mestdagh et al., 2022). M-path is an easy-to-use, closed-source platform with flexible ESM protocols, varied item formats, data collection and visualization, and just-in-time interventions, ideal for personalized research in clinical settings. m-Path's flexibility and support allowed us to implement a hierarchical account structure embedding therapists', supervisors', and patients' accounts under the research team. Patient data adheres to EU General Data Protection Regulation. Although m-Path visualizes patient data well, it lacks specific features for single case designs, like various trial designs or automated visual/statistical analysis. Q&C: *How will the participant flow be organized (especially, if a randomization is used in the design?) How will data analysis and visualization be conducted?*

#### Ethical Considerations and Data Protection

For all studies of the implementation process, the ethical approval is currently being obtained by the institutional ethics committee of the Department of Psychology at the RPTU Kaiserslautern-Landau. However, using the SCED infrastructure in routine clinical care and for research purposes comes with the challenge to meet requirements of treatment-related data and research data. For example, documentation and retention obligation apply to treatment-related data, research data may have to be deleted if the patient requests it. Specific in-depth interviews of Study 1 will be used to clarify questions regarding ethical considerations and data protection. An updated ethical approval will be obtained based on the results. Q&C: *Should there be a line between treatment-related data and research data? Can patients opt-out of using the SCED infrastructure and still receive psychological treatment for their condition? How is privacy secured including data gathering, transport, storage, analyses, and presentation?*

### Study 2: Development of SCED Procedure and ESM Protocol for Routine Clinical Care

The aim of the study is (1) to develop a SCED procedure for routine clinical care, (2) to develop an ESM protocol that describes the different parameters that need to be considered such as assessment duration, frequency, and sampling scheme, and (3) to find a balance between optimal and pragmatic study settings. The ESM protocol will be reviewed in discussion groups with a minimum of six psychotherapists and six patients as the stakeholders who are mostly affected by the ESM protocol (for a justification of the sample size see Study 1). The SCED protocol, along with the outcomes of the discussion groups, will be presented to the international advisory board and to the management board of the outpatient clinic to come to a final decision.

#### SCED Procedure

We created a working version based on the Risk of Bias in N-of-1 Trials (RoBiNT) scale, a critical evaluation tool to evaluate the methodological quality of intervention studies using single-case methodology (Tate et al., 2013). We outlined the procedure for routine clinical care and research projects that could use the infrastructure of the single-case clinic in the future. For routine clinical care, we plan to use a replicated ABCDE design in which EMA data is collected after the initial appointment (phase A: baseline), during up to five diagnostic sessions (phase B: diagnostic), and during the first treatment sessions (phase C: intervention). Whenever psychotherapists alter their treatment focus or change their therapeutic strategies, they can initiate a new intervention phase (phase C’, phase C’’, etc.). This allows them to directly continuously evaluate their respective therapeutic approach. Furthermore, ESM data will be collected after treatment has ended (phase D: posttreatment) and at 6-months follow-up (phase E: follow-up). Further details can be found in Table 1.

**Table 1:** Working version for the implementation of the single-case clinic based on the RoBiNT Scale (Study 2)

Item	RoBINT Scale	Suggestions for the implementation into routine clinical care	Possibilities for future research projects
*Internal validity subscale*			
1	Design	Replicated ABCDE Design A: baseline phase B: diagnostic phase C: intervention phase (C’, C’’, etc.) D: posttreatment phase E: follow-up phase	Replicated *randomized* designs Multiple baseline designs (concurrent and non-concurrent)
2	Randomization	No randomization due to practice-oriented focus	Randomization of treatment start per patient; randomization of treatment components (and their order)
3	Sampling behavior (all phases)	Baseline, diagnostic, posttreatment, and follow-up phase: 14 surveys per phase Intervention phase: open end	At least 5 data points per phase; patients with less than 3 data points per phase should be excluded from any statistical analysis
4	Blinding patient/psychotherapist	Blinding not possible and unethical	Blinding options are restricted in psychological treatments; in the case that interventions are compared against each other, neither patients, psychotherapists, nor supervisors should be informed about the research hypothesis
5	Blinding assessors	Blinding is not possible due to the use of mainly self-report measures; patients, psychotherapists, and supervisors are not blinded to the treatment phase	Blinding options are restricted in psychological treatments; for example, an independent statistician could be blinded to the treatment phase, although the time stamps (informative for data cleaning) will always show which data points belong to the baseline phase
6	Inter-rater reliability	Treatment progress will be monitored by the patient, psychotherapist, and supervisor; there will be no formal criterium, e.g. when to change the treatment strategy	Future studies might use additional assessment methods (e.g., video recordings, external ratings, passive sensing) that allow to assess inter-rater reliability
7	Treatment adherence	Adherence to CBT is secured by regular (video-based) supervision of psychotherapists (in training) by experienced psychotherapists (> 5 years’ work experience; completion of a specialized training for supervisors or otherwise accredited based on extensive requirements)	In future research projects, the existing infrastructure for video recordings could be used to formally check adherence via independent raters to a specific treatment protocol
*External validity and interpretation subscale*			
8	Baseline characteristics	Baseline characteristics (e.g., demographics, diagnoses, symptom severity) will be routinely assessed; during the diagnostic phase, the psychotherapist will conduct a functional analysis to develop a case conceptualization	Inclusion criteria could be chosen according to the respective research questions
9	Therapeutic setting	University-affiliated outpatient clinic in Germany (INCLUDE NAME AFTER PEER-REVIEW) in which bachelor graduates can obtain their master degree in psychotherapy and master graduates can obtain their license as psychotherapists with a special focus in CBT	Future studies could also use the SCED infrastructure to cooperate with other clinics
10	Dependent variable (target behavior)	Emotion regulation processes as a transdiagnostic mechanism; based on the functional analysis in the diagnostic phase, the psychotherapist and patients have the option to create additional individualized items to monitor the treatment progress	Dependent variables could be chosen according to the respective research questions
11	Independent variable (intervention)	The treatment starts with a diagnostic phase with a subsequent intervention phase; diagnostical tools and interventions will mainly be rooted in a CBT approach	Future studies could implement specific treatment manuals and protocols, as well as compare isolated treatment elements and interventions
12	Raw data record	Data sets for each individual patient will be recorded with an indication about which data points are missing; data will be stored while respecting data protection regulations	Future studies should strive to increase compliance of assessments (e.g., have regular compliance checks by research assistants)
13	Data analysis	Patients and psychotherapists are encouraged to look at the data collaboratively through visual inspection; psychotherapists will be encouraged to look at the data with their supervisors through visual inspection	Future research questions and their respective analytic approach should be preregistered in the OSF framework before beginning the statistical analyses
14	Replication	All patients and psychotherapists will be offered to the SCED infrastructure	One or two replications are considered good, more than three replications are considered optimal; with more and more data collected, future studies could divide a large data set into different subsets (e.g., training vs. test sample) to see whether the effect is replicated in different samples and determine cross-validation
15	Generalization	The posttreatment and follow-up phase intend to assess generalization effects; different patients will be treated by different psychotherapist and psychotherapists will be supervised by different supervisors	Generalization effect should be preferably monitored throughout treatment but the evaluation before and after treatment is also acceptable; for example, future studies could investigate whether treatment effect generalizes across skills (e.g., do skills to regulate negative emotion automatically translate in better skills to evoke and maintain positive emotions) or situations (e.g., do skills to regulate interpersonal situations automatically translate in better skills to regulation intrapersonal situations)

#### ESM Protocol

We followed systematic guidelines to consider different arguments when making methodological decisions (Janssens et al., 2018). A decision matrix was created that organizes different arguments along methodological questions (Supplemental Material, S3 Decision matrix for methodological questions, https://osf.io/54ewu). Arguments include the nature of the variable of interest, reliability and feasibility issues, as well as statistical requirements. Open methodological questions include study duration, measurement frequency, number of items, sampling scheme, instruction of items, and delay allowed to respond. The matrix was filled with typical practices or recommendations for ESM studies found in the literature (Eisele et al., 2020; Janssens et al., 2018; Wrzus & Neubauer, 2023). Based on the matrix, the research team formulated methodical suggestions for the SCED infrastructure regarding each methodological question. We decided to omit the issue of statistical requirements. Instead, future studies using the SCED infrastructure should discuss statistical requirements for the respective research question and analysis in a separate pre-registration in the Open Science Framework (https://osf.io/yex48/).

We propose to collect ESM data on 14 consecutive days during each SCED phase. We decided to not limit the study duration during the intervention phase with the aim to gather information about the extent that the daily surveys are used during treatment. Alternatively, a second (or more) 14-day assessment periods could be prompted during treatment (phase C’ or C’’ etc.). We suggest to collect one signal-contingent survey per day with the option of additional event-contingent surveys whenever the patient feels something important had happened. Each event-contingent survey will trigger a measurement burst with additional five surveys, randomly presented within the following hour. This measurement burst enables to monitor momentary mood on a microlevel. We decided to apply a semi-random sampling scheme with random beeps during an individualized time in the evening (e.g., 18-22 h). As delay allowed to respond, we suggest 60 min for the signal-contingent survey with a reminder after 30 min. For the event-contingent survey, we decided to close the survey after 30 min.

### Study 3: Development of the ESM Survey

This study aims (1) to identify a relevant and suitable clinical behavior and (2) to develop an ESM survey to assess this target behavior in routine clinical care. The survey will be part of our standard diagnostic procedure, allowing patients and psychotherapists to add individualized items if desired. The survey's specific content is less critical for implementing the SCED infrastructure and can be replaced or expanded in future research studies based on other variables of interest. However, it is a significant gap in SCED infrastructure implementation that validated ESM surveys are still limited (for a positive example see https://esmitemrepositoryinfo.com/). It is needed if it should be possible to aggregate the generated SCED data across patients (e.g., to investigate overarching research questions). We plan to follow recommendations in questionnaire development and validation, following sequential phases of item generation, scale development and scale validation (Boateng et al., 2018). During this process, a minimum of six stakeholders each will be consulted at several stages until information saturation is reached (for a justification of the sample size see Study 1). For example, patients will be involved via cognitive interviews to ensure the comprehensibility of the items (Darnall et al., 2017). Psychotherapists will be involved to ensure that the m-path data dashboard is used in a meaningful way for clinicians (Guest et al., 2006). Besides content validity, it will be crucial to assess its suitability as a SCED instrument, e.g. in terms of sensitivity to intrapersonal change (Lavefjord et al., 2021), and to consider the potential non-stationarity of the data (Ryan et al., 2023).

We propose to apply a twofold assessment strategy which yields the potential to understand the individual’s model in reference to what is normative (Wright & Zimmermann, 2019) and to capture complex change processes that are of greatest relevance to individual clients being also most consistent with the clinical reality of psychotherapeutic work (Lloyd et al., 2019).

#### Emotion Regulation

As a first draft of an ESM survey, we decided to focus on emotion regulation as an important transdiagnostic mechanism to complement the thus far primarily symptom-oriented diagnostics with a mechanistically informed approach. Emotion regulation refers to the ‘ability to modulate the intensity, frequency, and duration of positive and/or negative emotions’ (Boemo et al., 2022, p. 1). It is a dynamic, multi-stage process (Gross, 2015), making it well-suited for ESM measurement. Emotion regulation is crucial in psychological disorder development and maintenance (Fernandez et al., 2016). Understanding its complexity in everyday life can reveal regulation difficulties and inform targeted therapeutic interventions (Aldao et al., 2015; Gross & Jazaieri, 2014). Moreover, emotion regulation could be studied more rigorously as mechanism of change in psychotherapy (Palmieri et al., 2022). We plan to include the following emotion regulation processes in the ESM survey: momentary affect (Cloos et al., 2022), information on the appraisal of specific situational contexts (Doré et al., 2016), emotion regulation strategies (Boemo et al., 2022), emotion regulation motives (Tamir, 2016), and emotion regulation success (Gruber et al., 2012). This would allow a differentiated assessment of an important transdiagnostic mechanism within and across patients. Data provides important insight for psychotherapy independent from specific diagnosis and could still be aggregated across patients.

#### Idiographic Items

Above that, two idiographic items are formulated, a problem-focused and goal-focused idiographic measure. Idiographic measures offer personalized insights and empower clients (Elliott et al., 2016; Wright & Zimmermann, 2019). Especially, goal-setting has been shown to have effects of *d* = 0.34-0.40 of its own (Epton et al., 2017; Harkin et al., 2016). However, the item generation is not without challenges (Sales et al., 2023). The identification of relevant problems and goals is a difficult task as patients might not always be able to report their problems and goals in clear and precise manners. Also, problems and goals might change throughout the process of psychotherapy. For those reasons, we plan to build upon existing measures. According to two recent systematic reviews (Lloyd et al., 2019; Sales & Alves, 2016) examples for short problem-focused measures that could serve as a starting point are the Simplified Personal Questionnaire (PQ; Elliott et al., 2016; Shapiro, 1961) and the Psychological Outcome Profiles (PSYCHLOPS; Ashworth et al., 2005) and goal-focused measures are Goal Attainment Scaling (Kiresuk & Sherman, 1968), Goals Form (Cooper & Xu, 2023), and Youth TOP Problems (Weisz et al., 2011).

### Study 4: Testing Usability and Feasibility

**Fig. 3:**
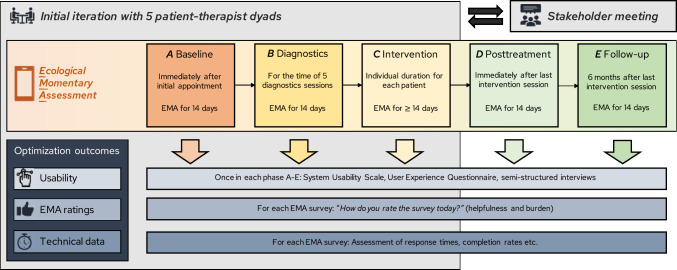
Design and process of the optimization phase

In the *optimization phase*, the aim is to test usability and feasibility and to customize the SCED infrastructure accordingly. We plan to carry out several SCED iterations using a replicated ABC(DE) design (see Table 1; Tate et al., 2013). Each iteration will comprise 6–12 patient-therapist dyads (Bartels et al., 2022). To accelerate the optimization process, only phases A, B, and C are initially included to identify critical points of improvement. Phases D and E will be carried out, but they will not be decisive for the optimization process. Based on the draft of the ESM protocol, the respective phases should comprise 14 assessment days. Patient-therapist dyads are informed that participation also includes a subsequent stakeholder meeting. The results of each iteration will be presented at the respective stakeholder meeting. Stakeholders will collaboratively identify areas for improvement in each component of the infrastructure. Based on this, they may choose to initiate further rounds of iterations. The optimization loop continues until stakeholders agree that the infrastructure is ready for the implementation phase. Decisions will be consensus-based, requiring agreement from all stakeholders. Figure 3 presents an overview about the design and process of the optimization phase.

#### Usability Measures

A mixed methods approach will be used to determine the usability of the SCED infrastructure, including questionnaires, technical data, and semi-structured interviews (see Table 2). Questionnaires will be administered at the end of each SCED phase with the concluding assessment comprising questionnaires and a semi-structural interview 6 weeks after the intervention started. To facilitate its evaluation, we decomposed the infrastructure into the following components: initial expectations, the onboarding process, the practical use of the m-path app, the feasibility of the ESM protocol and survey (including possible negative side effects), the clarity of the visual dashboard, the utility for clinical practice, and data management aspects.

**Table 2:** Usability Measures (Study 4)

Questionnaires	ESM data	Semi-structural interview
• Adapted System Usability Scale (SUS) (Brooke, 1996; Lyon et al., 2020) to evaluate the usability of the m-path app (e.g., *‘I think I would like to use this app frequently.’*) and dashboard with the visual presentation of the ESM data (e.g., *‘I think I would like to use this dashboard frequently.*’) on a 5-point Likert Scale (1 = strongly disagree to 5 = strongly agree) • User Experience Questionnaire (UEQ-S) (Schrepp et al., 2017) to assess the subjective impression of users towards the user experience of the m-path app on a 7-point Likert scale (-3 = fully agree with negative term) to + 3 = fully agree with positive term) grouped into 6 subscales: *attractiveness, perspicuity, efficiency, dependability, stimulation, novelty*	• Patients have to rate of how burdensome (0 = neutral to 10 = disturbing) and helpful (0 = neutral to 10 = helpful) each survey is *(‘How do you rate the survey today?*’) • Response times, compliance rates, and indicators of careless responding will be retrieved from the ESM surveys • ESM data will be merged with data routinely collected at the outpatient clinic (such as clinical diagnosis, severity of symptoms, and sociodemographic data); difficulties emerging during this process will be recorded in a protocol to be able to resolve the issues for future iterations	Interview questions are intended to cover different components of the SCED infrastructure: • Initial expectations (e.g., *‘What did you expect at the beginning? Have these expectations been met?’*) • Onboarding process (e.g., *‘How can we improve the onboarding process?’*) • Practical use of the m-path app (e.g., *‘How did you get on with the m-path app?’*) • Feasibility of the ESM protocol and survey (e.g., *‘Would you change anything about the survey, including the frequency of assessment?’*) • Negative side effects (e.g., *‘Did you experience any negative side effects? How could negative side effects be reduced?’*) • Clarity of the dashboard (e.g., *‘Did you use the dashboard? What would you like to change about the dashboard?*’) • Utility for clinical practice (e.g., *‘Was the app part of the treatment/supervision? What could be changed to maximize its utility?’*)

Note. Usability measures will be administered to both patients and psychotherapists after each SCED phase; supervisors will also be invited to rate the System Usability Scale (SUS) to evaluate the dashboard (i.e., the visual presentation of the data) and conduct the semi-structural interview

#### Feasibility Measures

Feasibility is evaluated along the outcome domains of implementation research that are commonly used in feasibility studies (Arain et al., 2010; Proctor et al., 2011): *Acceptability* and *appropriateness* are assessed as part of the usability measures. Feasible *adoption* is reflected in participation (≥ 50% offered using the SCED infrastructure will enroll in the study and ≥ 70% of the participants who enrolled will complete participation; Frumkin et al., 2021). Reasons for drop-out will be assessed if participants consent to provide information. In addition, *penetration, compliance* and *retention* will be monitored.

### Study 5: Piloting the Implementation of the SCED Infrastructure

The study aims (1) to determine how users accept, perceive, adopt, and integrate the SCED infrastructure into routine clinical care and (2) to use results to initiate strategies for further improvement. For that reason, the SCED infrastructure will be offered in routine clinical care to generate first evidence regarding perceptual and behavioral implementation domains. We intend to offer all new admissions to our clinic to use the SCED infrastructure for a 1-year period. Both patients (irrespective of their clinical diagnosis except for acute suicidal tendencies and psychotic symptoms) and psychotherapists will be invited to participate in the implementation study. We expect to have approximately 250 new admissions in a 1-year period. On average, during the last five years we had 295 new admissions per year, but due to the change of the system we expect a slight decrease. In 2021 and 2022, we had 77 active psychotherapists and 72 respectively. The number of patients and psychotherapists who decide to use the infrastructure will be used to estimate the adoption rate as one implementation outcome (see Section “Implementation Outcome Measures” and Table 3 for more information).

**Table 3:** Implementation Outcome Domains and Measures (Study 5)

Domain	Definition	Concrete Operationalization	Measure	Criteria
Acceptability and Appropriateness	Acceptability is defined as the perception among different stakeholders that an innovation is agreeable, palatable, or satisfactory Appropriateness refers to the perceived fit, relevance, or compatibility of an innovation for a given practice or setting	Degree that patients and psychotherapists think the SCED infrastructure is acceptable and helpful to improve clinical practice in an outpatient setting	(1) The tool (i.e., the handling of the m-path app) and dashboard (i.e., the visual presentation of the data) are rated independently on a 5-point Likert scale (1 = ‘strongly disagree’ to 5 = ‘strongly agree’): • *‘The tool is acceptable for clinical practice’* • ‘*The tool is helpful for clinical practice’* • *‘The dashboard is acceptable for clinical practice’* • ‘*The dashboard is helpful for clinical practice’* (2) Three subscales of an adapted version of the Adoption of Information Technology Innovation Measure (Moore & Benbasat, 1991) are rated on a 7-point Likert Scale (1 = ‘extremely disagree’ to 7 = ‘extremely agree’): • Relative advantages (e.g., *‘Using the tool improves the quality of the treatment’*), • Compatibility (e.g., *‘Using the tool is completely compatible with my current situation’*) • Ease of use (e.g., *‘Overall, I believe that the tool is easy to use’*) (3) Adapted version of the Evidence-based Practice Attitudes Scale (EBPAS; Aarons, 2004) together with its extension (Aarons et al., 2012)	(1) Mean scores of > 3.5/5 on each item are considered sufficiently acceptable and appropriate (2) Mean scores of > 4.5/7 on each subscale are considered sufficiently advantageous, compatible, and easy to use (3) N/A
Adoption	Adoption comprises the intention, intentional decision, or action to try or employ an innovation	Number of individuals who are willing to use the SCED infrastructure (i.e., who consent to test the infrastructure as part of the study)	(4) Number of patients and psychotherapists who decided to use the infrastructure divided by the number of invitations (i.e., new admissions to the clinic)	(4) A rate ≥ 50% is considered substantial demand
Penetration	Penetration refers to the integration of a practice within a setting	Number of patients and psychotherapists who actually use the SCED infrastructure (e.g., in terms of responding to the ESM surveys; retrieving and discussing the dashboard during treatment or supervision)	(5) ESM data (including signal- and event-contingent surveys of completed ESM surveys (i.e., the last question has been answered)) is used to derive an estimate for the penetration: • Exploration of arithmetic values (e.g., median, range, mean standard deviation) for the number of completed ESM surveys for each SCED phase, especially for the open-ended intervention phase • RoBiNT scale requires each phase to consist of at least five, but not less than three data points for the analysis of SCED data (Tate et al., 2013). We will evaluate each phase separately, with ≥ 5 data points as an acceptable criterion. For each phase, we divide the number of patients who fulfill the acceptable (≥ 5 data points) criterion by the number of participating patients (6) Indication how often patients and psychotherapists retrieved the dashboards either to prepare for treatment or discuss the dashboard during treatment on a 5-point Likert scale (1 = never to 5 = every session) after each SCED phase. Psychotherapists will also be asked to indicate how often they discussed the dashboard with their supervisors • *‘How often did you retrieve the dashboard (e.g., to prepare for treatment or discuss the dashboard during treatment)?’* • *‘How often did you discuss the dashboard together with your supervisor?’*	(5) A rate ≥ 75 is considered substantial use (6) Mean scores of > 3.5/5 on each item are considered substantial use
Implementation costs	All costs needed for an implementation effort	All monetary resources needed for the implementation of the SCED infrastructure. These costs could either be anticipated (e.g., for the m-path software) or unexpected (i.e., they only become clear in the course of implementation)	Implementation costs are also difficult to calculate because they vary substantially in the different settings (7) A list of the costs we have incurred (e.g., required working time of an employee to coordinate the single-case clinic)	
Compliance and Retention Rates	Compliance refers to the ratio of completed surveys over the theoretical maximum number of surveys Retention refers to the proportion of participants included in the final analyses	Compliance and retention rates to the ESM surveys intend to evaluate to what extent the data generated in a routine clinical setting could be used for future data analyses to address overarching research questions	(8) Ratio of completed signal-contingent surveys divided by the number of theoretically possible surveys (as an indicator for compliance) (9) Proportion of participants who responded to at least five of the signal-contingent surveys after data cleaning (as an indicator for retention)	(7) Rates of 70–80% are considered satisfactory compliance (8) Rates of 90% are considered satisfactory retention

Note. Definitions are derived by Proctor et al. (2011). Implementation outcome measures will be administered to both patients and psychotherapists after each SCED phase; only the Evidence-based Practice Attitudes Scale (EBPAS) will be administered once at the study beginning (i.e., when patients and psychotherapists decide whether to use the SCED infrastructure); this will allow to compare individuals who are willing to use the SCED infrastructure with those who are not. Criteria for a successful implementation were chosen according to previous research (Schleider et al., 2020); criteria for compliance and retention rates were formulated based on systematic reviews (Rintala et al., 2019; Vachon et al., 2019; Wrzus & Neubauer, 2023); it should be noted, however, that these values were estimated in the context of research studies (as opposed to a routine clinical care setting)

To evaluate our implementation process, we assess several perceptual and behavioral outcome domains (Proctor et al., 2011). We hope to obtain some outcome measures from people who are willing to use the SCED infrastructure, as well as those who are not interested. This will allow us to conduct some exploratory analyzes about how these groups differ. For patients, we expect to collect a relatively diverse sample which will probably allow such a comparison.

Results of the initial implementation study will be a first indicator for the expected sustainability of the SCED infrastructure in our clinic. In case, results do not meet our predefined criteria for a successful implementation, the research team will develop a proposal for another implementation strategy plan (Kirchner et al., 2020b; Powell et al., 2015) to maintain and further improve the uptake of the SCED infrastructure. Other implementation strategies could comprise the provision of interactive assistance or the training of stakeholders. Again, the implementation plan will be optimized and finalized together with different stakeholders. In either case, we plan to replicate the pilot implementation study for another year, with the goal to either test whether implementation outcomes substantially improve compared to the first implementation round or to evaluate its sustainability.

#### Implementation Outcome Measures

Perceptual and behavioral outcome measures were selected in accordance with a generally recognized taxonomy for outcome domains in implementation research (see Table 3; Proctor et al., 2011). For our study purposes, we considered the outcome domains acceptability, appropriateness, adoption, and penetration. In addition, we plan to report on compliance and retention rates as indicators inherently linked to ESM studies. Criteria for a successful implementation were chosen according to previous research (Schleider et al., 2020). Criteria for compliance and retention rates were formulated based on systematic reviews (Rintala et al., 2019; Vachon et al., 2019; Wrzus & Neubauer, 2023). We decided to omit the domains of feasibility, fidelity, and sustainability as these issues are addressed in the other phases of the agile research framework. For example, feasibility will be ameliorated and tested in phase 3, while sustainability will be addressed in phase 5. Likewise, it is difficult to include fidelity in an agile research framework as methodological decisions can change based on outcomes from previous phases. We have chosen behavioral measures whenever possible. Given the general lack of unified implementation measures with mostly unknown psychometric quality, questionnaires to assess perceptual outcomes were selected based on a recent systematic review evaluating the psychometric properties of implementation measures (Mettert et al., 2020). Perceptual implementation outcome measures will be collected from both patients and psychotherapists when they decide whether they want to use the SCED infrastructure prior to treatment. Perceptional implementation outcomes will also be administered after each SCED phase to record their development over time. In the intervention phase, implementation measures will be administered 6 weeks after the intervention started. If the post-treatment and follow-up phases are not yet available for all patients at the time of data analysis, patients will be invited to take part in another 14 ESM assessment days and to provide concluding information on perceptual implementation outcome measures.

### Study 6: Implementation of the SCED Infrastructure

The aim of the study during the *implementation phase* is to evaluate the SCED infrastructure compared to routine diagnostic procedures prior implementation. A quasi-experimental single case AB-design with the outpatient clinic as entity will be conducted. The design has the advantages that it reduces confounders (e.g., secular trends, seasonality) and allows to measure long-term effects (Bernal et al., 2017). We will use a continuous sequence of outcome data taken repeatedly over time from the outpatient clinic prior implementation and compare it to outcome data post implementation. This time series is used to estimate an interrupted time series regression. An underlying trend is established, which is ‘interrupted’ by the implementation of the SCED infrastructure at a known point in time (Bernal et al., 2017). Therefore, pre- and post-implementation need to be clearly distinguished (Miller et al., 2020). A graphical example of an interrupted time series is presented as Supplemental Material (S1 Design illustrations, https://osf.io/dkytg).

In our case, data are considered for the pre-implementation phase up to the start of Study 5 and for the post-implementation phase after the completion of Study 5, when the SCED infrastructure is introduced for everyone into routine clinical care. Two items assessing satisfaction and burden of the diagnostic procedure (that includes the ESM assessments after the implementation of the SCED infrastructure) will be used to evaluate the new diagnostic procedure. In addition, we will take an exploratory look at clinical effectiveness using the Symptom Checklist – 90 (Derogatis & Unger, 2010; Franke, 2014). We expect a slight level and slope change regarding clinical effectiveness because research has shown that self-monitoring already effects outcomes positively leading to level change (Bartels et al., 2019; Guo & Albright, 2018; McBain et al., 2015; Simons et al., 2015). In addition, the continuous feedback might reduce treatment failure and improve the positive effects of psychotherapy for example through enhanced tailoring (Lambert & Harmon, 2018; Lutz et al., 2019), leading to a temporary slope change resulting in a level change.

## Discussion

SCEDs are prospective idiographic research designs aiming to estimate meaningful treatment effects for individual patients (Vlaeyen et al., 2020). We argue that the method is highly suitable to foster the development and evaluation of personalized psychological treatments for two main reasons: First, current data-driven personalization efforts are mainly based on nomothetic data that come with limitations due to power (Lorenzo-Luaces et al., 2021) and due to invalid inferences to the individual. In contrast, the SCED infrastructure offers a truly idiographic approach to research and practice. Nevertheless, generalization is enabled through replication creating data that can also be accumulated to answer nomothetic research questions. Second, since the SCED infrastructure allows systematic manipulation, it is valid to draw causal inferences and estimate treatment effects for the individual patient which extends the observational character of current routine outcome monitoring systems. For those reasons, we outlined the step-by-step implementation with stakeholder involvement of a SCED infrastructure including ESM into routine clinical care of a German outpatient research and training clinic. While focusing on routine clinical care in this protocol, the infrastructure can also serve future research studies using SCED methodology.

To our knowledge, such a SCED infrastructure has not been implemented in the context of mental health up till now. Yet, such an infrastructure could be associated with several advantages: The SCED infrastructure could link everyday life to the psychotherapy process more closely and brings the psychotherapy process back into daily life. For example, it could empower patients through self-monitoring and feedback and enhance clinical and collaborative decision-making through continuous evaluation (Herzog et al., 2022; Schemer et al., 2022), also overcoming biases of clinical judgements (Kaiser et al., 2022a, 2022b). Therapists will probably find this feedback highly credible because it results from multiple assessments in the daily life of patients. The feedback provides new information about the patients' progress with high precision. This accurate information from a credible source facilitates the integration of feedback and update of beliefs about patients’ therapy progress—a necessary prerequisite for the feedback to effect treatment outcome (Herzog et al., 2023).

Practitioners in routine clinical care could also use systematic manipulation in an ongoing treatment to evaluate their own clinical practice for their particular patients (Piccirillo & Rodebaugh, 2019). Thus, the infrastructure could bring science into practice allowing a truly individualized and at the same time evidence-based treatment to deliver the most effective treatment for the respective patient. Above that, it provides an infrastructure for a range of idiographic and/or nomothetic research questions that need intensive longitudinal data assessment and/or systematic manipulation: What specific emotional regulation difficulties does the individual patient have and how can they best be addressed in therapy? How effective is a particular treatment strategy such as high-intensity exposure for an individual patient or specific patient groups? To what extent does a change in therapeutic strategy lead to an improvement in the process outcome if there was no improvement or even a deterioration in the previous phase? Asked differently, how does changing the therapeutic strategy too quickly affect the therapeutic process? How valid are inferences from group data to the individual vs. from individual data to the group regarding the psychotherapy process (ergodicity problem; see Adolf & Fried, 2019; Fisher et al., 2018)? Methodologically, the SCED infrastructure allows to focus on patterns within a person using intensive longitudinal data that can be used to inform decisions about personalized treatment strategies for this specific person (Delgadillo & Lutz, 2020). For example, it might be interesting to take into account the dynamic nature of psychological mechanisms. Once data has accumulated, personalized advantage indexes could be developed to guide personalized treatment selection on a very fine-grained level (DeRubeis et al., 2014).

The step-by-step implementation of a SCED infrastructure including EMA with stakeholder involvement comes with the following limitations: First, the step-by-step implementation is initiated expecting that the SCED infrastructure will enable studies that will push personalization forward leading to more effective treatments for individual patients. However, evidence that will support this expectation can only be generated after implementation. Therefore, we did not describe a stopping rule for the implementation process, even though there could be a high patient burden, lacking penetration, or it might not lead to better treatment in the long run.

Second, it must be kept in mind that evidence for the effectiveness of stakeholder engagement from experimental or empirical studies is lacking (Slattery et al., 2020). At the same time, it comes with an increase in research resources such as time, costs, and training efforts, changes that might not be feasibly and uncertainty to resolve conflicts (Holzer et al., 2022; Tindall et al., 2021). Further barriers are the use of technical jargon, power imbalances between the researcher and stakeholders, difficulty for stakeholders to understand how their input is reflected in the final research (Holzer et al., 2022). Therefore, it is important to introduce the research purpose, the questions being addressed and the requirements for the studies carefully (Holzer et al., 2022). Role descriptions and responsibilities should be clearly defined and the stakeholders’ time and input should be valued to build trust and rapport between researchers and stakeholders (Slattery et al., 2020).

Third, there are many obstacles when implementing a new infrastructure into an existing routine. As the consolidated framework for implementation research outlines, many domains need to be taken into consideration including the inner and outer setting, the individuals involved in the implementation, and the implementation process itself (Damschroder et al., 2009, 2022). While this protocol outlines the implementation process and some aspects of the other domains, the adaptation to the inner and outer setting will present numerous challenges including the accounting of the service with insurances, patient data protection and handling policies, or the maintenance of the service with existing staff. Even though we try to address these challenges from early on, most of them will come up throughout the process. The implementation strategies were chosen to respond to those upcoming issues flexibly on the way.

Despite these limitations, we are optimistic that challenges can be faced and overall, the potential of the SCED infrastructure will outweigh the burden. We will propose a business model for a Outpatient Clinic for Personalized Psychotherapy—a learning mental health care system that can reduce the research-practice gap. We will implement this SCED infrastructure in combination with EMA into routine clinical care step-by-step with stakeholder involvement to leverage the potential of person-specific data-based and data-driven methods (Schemer et al., 2022).

## Supplementary Information

Below is the link to the electronic supplementary material.

Supplementary file1 (PDF 118 kb)

Supplementary file2 (PDF 62 kb)

Supplementary file3 (PDF 70 kb)

## Footnotes

1 Different terms are used in the context of SCEDs such as Single-Case Experimental Design (SCED), Single-Subject Research Design, Single-Case Observational Design (SCOD), N-of-1 trial, Single Patient Open Trial (SPOT) and Single-Case Design (SCD) (Krasny-Pacini & Evans, 2018; Nikles et al., 2021). Although SCEDs is widely used, we use from here on SCD as an umbrella term, allowing also observational and open designs and thus fitting better with the intended use in our routine clinical care setting.

2 Experience Sampling Methodology (ESM), Ecological Momentary Assessments, and Ambulatory Assessments comprise data collection methods used to study individuals’ daily life experiences and environmental features in naturalistic settings (see Myin-Germeys & Kuppens, 2021). In particular, ESM and Ecological Momentary Assessment prompt individuals to respond to surveys assessing psychological constructs as they unfold in daily life (Schemer et al., 2022). For consistency reasons, we use the term “ESM”.
