# Supplementary material for: Leveraging Single-Case Experimental Designs to Promote Personalized Psychological Treatment: Step-by-Step Implementation Protocol with Stakeholder Involvement of an Outpatient Clinic for Personalized Psychotherapy
Source: Adm Policy Ment Health. 2024 Mar 11;51(5):702–24. doi: 10.1007/s10488-024-01363-5 (PMC11379774; doi:10.1007/s10488-024-01363-5)
Supplement: Supplementary file 1 — Supplementary file1 (PDF 118 kb) [file 10488_2024_1363_MOESM1_ESM.pdf]

Examples of Single Case Designs

Different Single Case Designs exist (Kazdin, 2019; Kratochwill et al., 2013; McDonald et al., 2017):

Figure 1 presents two examples of SCED designs: Figure 1a illustrates an observational design. It is characterized by repeated measurements of a behavioral outcome over time within one individual (McDonald et al., 2017). In our case, it could present the data from an individual who documented emotion regulation skills in a daily diary over the course of psychotherapy. The skills increased over time. This is the design we plan to use in routine clinical care.

Figure 1b presents a basic version of an experimental ABAB design. The effects of an intervention are evaluated by contrasting a baseline condition without intervention (Phase A, e.g., waiting time) with an intervention condition (Phase B, e.g., intrapersonal emotion regulation skills). Both phases are repeated to control for third variable contributions to the effect. If the effect improves during the first intervention phase, drops as intervention is withdrawn (e.g., because the new behavior is not yet well-established and emotion regulation in interpersonal contexts remains a challenge), and increases again during the second intervention phase (e.g., targeting interpersonal emotion regulation in addition), it is likely due to the intervention (Kazdin, 2019). This design helps researchers establish a causal relationship between the intervention and the observed effect by ruling out alternative explanations related to third variables. In our case, it could be used in future research projects that aim to evaluate specific interventions systematically. It is possible to look at different outcome variables that are ideally based on a specific maladaptive mechanism or the target variable of an intervention. In the example above, two different outcomes could have been used to differentiate the evaluation of intra- and interpersonal emotion regulation skills. It should be noted, however, that the change processes are usually not that clear-cut and the figure is for illustrative purposes only.

Figure 1

a) Exemplary visualization of an ABCD design

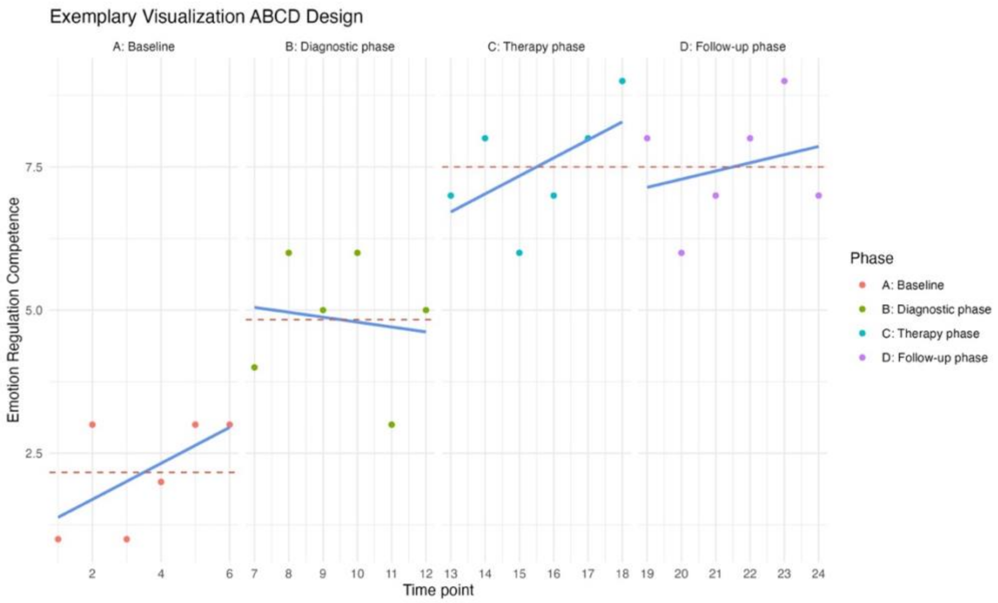

b) Exemplary visualization of an ABAB design

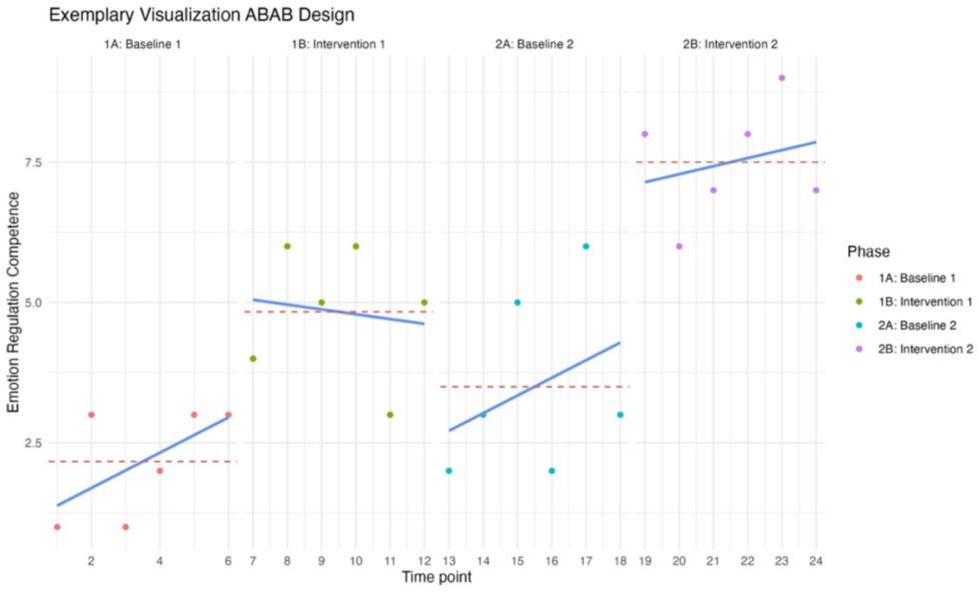

Note. The dots represent the indicated values of the emotion regulation competence. The dashed red line represents the mean of the emotion regulation competence in each phase. The blue line represents a linear trend of the emotion regulation competence in each phase.

Example of an interrupted time series

Figure 2 illustrates the interrupted time series showing an 11% [relative risk (RR) 0. 894; 95% confidence interval (CI) 0.864-0.925; P < 0. 001) decrease of hospital admissions for acute coronary events when the smoking ban was implemented in Sicily.

Figure 2

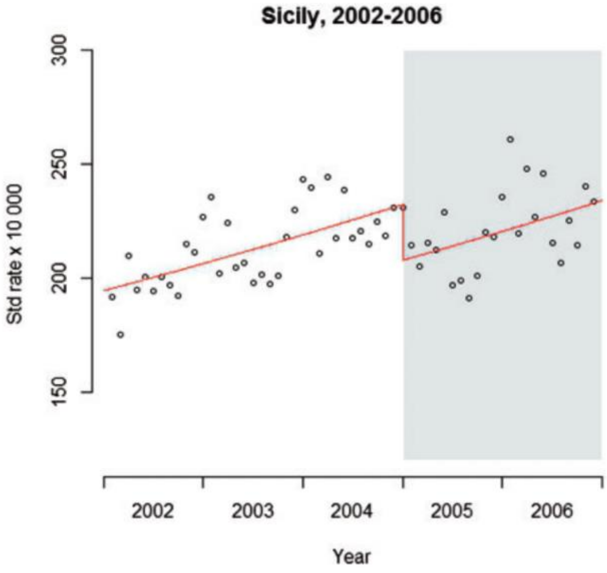

Note. Interrupted time series with level change regression model. The line indicates the predicted trend based on the unadjusted regression model. The figure is reprinted with permission of the authors (Bernal et al., 2017).
