# Supplementary material for: Leveraging Single-Case Experimental Designs to Promote Personalized Psychological Treatment: Step-by-Step Implementation Protocol with Stakeholder Involvement of an Outpatient Clinic for Personalized Psychotherapy
Source: Adm Policy Ment Health. 2024 Mar 11;51(5):702–24. doi: 10.1007/s10488-024-01363-5 (PMC11379774; doi:10.1007/s10488-024-01363-5)
Supplement: Supplementary file 2 — Supplementary file2 (PDF 62 kb) [file 10488_2024_1363_MOESM2_ESM.pdf]

Essential stakeholders and their functions

Stakeholder	Function	Identification
	Users	
Patients and their family members	Enter their own data<br>Access their own data<br>View and interpret results	Patients of the outpatient clinic
Therapists	Manage their patients<br>Create items and set up questionnaires, ESM protocols, and SCDs<br>Monitor data collection progress<br>Intervene (if needed)<br>View and interpret results	Therapists in training;<br>Licensed therapists
Supervisors	Manage their patients' data<br>View and interpret results	Supervisors of the outpatient clinic
Clinical researchers	Create items and set up questionnaires, ESM protocols, and SCDs<br>Implement SCD, including:<br>Intervention (if needed)<br>Capturing deviations and adjustments to the protocol<br>Monitoring unexpected events<br>Assessment of fidelity measures and interrater-reliability	PhD students<br>Postdocs<br>Professors
	User support	
Statisticians	Review SCD and collected data for validity and/or aggregate analysis<br>Download identified or de-identified data for offline analysis<br>Run statistical analysis	(Consulting) Statisticians of the research group
Administrative team	Institutional oversight and management<br>Support the implementation	Student assistants<br>Staff of the Outpatient Clinic
System administrators	Create user accounts<br>Support the operation of the IT system<br>Provide user tech support	Student assistants<br>m-Path tech support team
Developers	Problem-solve operational code<br>Develop new features	m-Path team
Healthcare delivery system	Institutional oversight and management<br>Provides patient flow<br>Provides management and facilities	Staff of the outpatient clinic
Regulatory agencies	Provide ethical guidance in the implementation process	Institutional Review Board of the University
	Collaborators	
Scientific Advisory Board	Support scientifically the implementation process<br>Anticipate and trouble-shoot problems	Leading experts in the field of SCD
Training staff	Train therapists (and supervisors) in SCD methodology<br>Train therapists (and supervisors) to use m-Path	Senior lecturers and training staff of the outpatient clinic
