# Supplementary material for: Leveraging Single-Case Experimental Designs to Promote Personalized Psychological Treatment: Step-by-Step Implementation Protocol with Stakeholder Involvement of an Outpatient Clinic for Personalized Psychotherapy
Source: Adm Policy Ment Health. 2024 Mar 11;51(5):702–24. doi: 10.1007/s10488-024-01363-5 (PMC11379774; doi:10.1007/s10488-024-01363-5)
Supplement: Supplementary file 3 — Supplementary file3 (PDF 70 kb) [file 10488_2024_1363_MOESM3_ESM.pdf]

S2 Example decision matrix to address methodological questions based on different levels of argumentation

	Nature of the variable	Representativeness	Feasibility	Suggestion
**Study duration (days)**	14 days as common time criteria for psycho-pathology	Stability criteria: Capture typical patterns in daily life and detect reliable change processes<br><br>Median: 17 days; IRQ: 7 – 30 days (Janssens et al., 2018)<br><br>Median: 7 days; range: 2-180 (Wrzus & Neubauer, 2023)	Reduce participant burden	Baseline, diagnostic, and posttreatment phase: 14 days<br>Intervention phase: open end
**Measurement frequency (times/day)**	Dynamic nature of emotion regulation processes	Median: 5x/day; IQR: 3 – 10x; range: 1 – 50x (Janssens et al., 2018)<br><br>Median: 7x/day; range: 1.7-81x (Wrzus & Neubauer, 2023)	Sampling frequency (3x, 6x, or 9x/day during 14 days) had no effect on perceived burden and data quantity (Eisele et al., 2020)	1x per day<br>Additional event-contingent surveys with measurement burst (that is five further surveys randomly presented within the following hour to monitor momentary mood)
**Number of items**	Complexity of emotion regulation processes discussed in the emotion regulation literature	Median: 30 items; IQR: 19 – 55 items (Janssens et al., 2018)	30-item ESM questionnaire was associated with lower burden and better data quantity compared to 60-item ESM questionnaire (Eisele et al., 2020)	We aim to reduce the item number, also shortening and simplifying their language and highlighting optional items, while recognizing the complexity of emotion regulation processes.

**Sampling scheme**<br>**Fixed**<br>**Random**<br>**Semi-random**	Random sampling scheme increases the representativeness of the data and avoids reactivity or anticipation effects	Semi-random sampling scheme gained popularity in recent years (Janssens et al., 2018)	Fixed time easier to remember for patients	Semi-random: random beep during an individualized time in the evening (e.g., 18-22h)
**Item instruction**<br>**Momentary**<br>**Retrospective**<br>**Both**	Assessment of emotions in specific daily contexts	Majority of researchers use a combined momentary and retrospective assessment design (Janssens et al., 2018)		Momentary assessment of affect<br>Retrospective assessment based on specific emotion-triggering situations
**Delay allowed to respond (minutes)**	Triggering situations change over time	Median: 30min; IRQ: 15 – 60min; range: 1.5min – 24h	Reduce missing values	Daily survey: 60min (reminder after 30 min)<br>Event-contingent survey: 30min

Note. The example decision matrix is filled with typical practices or recommendations found in previous ESM studies
